# Unveiling the gender gap in research: a bibliometric analysis of the 100 most-cited articles on food-borne pathogen outbreaks from 1990 to 2020

**DOI:** 10.3205/dgkh000467

**Published:** 2024-03-05

**Authors:** Bisal Naseer, Rawal Alias Insaf Ahmed, Mohsan Ali, Muhammad Talha, Saad Azizullah, Amar Anwar

**Affiliations:** 1 King Edward Medical University, Lahore, Pakistan; 2 Provincial Disease Surveillance & Response Unit, Hyderabad, Sindh, Pakistan; 3 Combined Military Hospital Medical College, Lahore, Pakistan; 4 Health Department, Government of Sindh, Sindh, Pakistan

**Keywords:** women representation, food-borne diseases, outbreak investigations

## Abstract

**Introduction:**

Despite a recent increase in the representation of female authors in scientific literature, a significant gap persists concerning the inclusion of women in research. This necessitates the analysis of published literature from a gender perspective. This study aimed to provide gender distribution in authorship in the 100 most-cited articles on food-borne pathogen outbreaks from 1990 to 2020.

**Methods:**

Bibliometric analysis was conducted using the Scopus database. Two reviewers were selected to search the database. We included the 100 most-cited articles on foodborne outbreak investigations. The analysis was conducted using Statistical Package for Social Sciences (SPSS) version 26 and Microsoft Excel version 2016. The citation data, including total citations, citations per year, and representation of women as first and senior authors, was analyzed in terms of frequencies, mean, median, and interquartile range. The correlation between journal impact factor and the representation of women in high-impact factor journals was determined. A p-value of <0.05 was considered significant.

**Results:**

Most of the top-cited articles were published between 2001 and 2010 (n=47). The top 3 most-cited articles were from the USA. Of the total 100 articles, women were the first and last authors in 46% and 28% of the articles, respectively, reflecting a significant gender gap. However, the proportion of females as principal investigators gradually increased from 25% (n=10/30) to 52% (n=24/47) during the period 2001–2010 and to 92% (n=12/13) during 2011–2020. The USA had the highest number of included articles (n=48), and women were principal authors in 56% (n=27) of them. The lowest representation of women was observed in Austria, Denmark, Japan, Netherlands, New Zealand, Nigeria, Portugal, and the United Kingdom.

**Conclusion:**

Women are under-represented in published literature on food-borne pathogen outbreaks. Although the representation of women as principal authors has recently increased, disparities still exist at the senior-author level, calling for women’s advancement in academic science.

## Zusammenfassung

**Einleitung:**

Obwohl der Anteil von Frauen in der wissenschaftlichen Literatur in letzter Zeit zugenommen hat, besteht nach wie vor eine erhebliche Lücke bei der Einbeziehung von Frauen in die Forschung. Das macht eine Analyse der Literatur aus der Geschlechterperspektive erforderlich. Ziel dieser Studie war es, die geschlechtsspezifische Verteilung der Autorenschaft in den 100 meist zitierten Artikeln zu lebensmittelbedingten Ausbrüchen im Zeitraum 1990–2020 zu ermitteln.

**Methode:**

Die bibliometrische Analyse wurde mit Hilfe der Datenbank Scopus durchgeführt. Zwei Reviewer wurden mit der Suche in der Datenbank beauftragt. Es wurden die 100 am häufigsten zitierten Artikel zur Untersuchung lebensmittelbedingter Krankheitsausbrüche berücksichtigt. Die Analyse wurde mit SPSS, Version 26, und Microsoft Excel, Version 2016, durchgeführt. Die Zitationsdaten einschließlich der Gesamtzahl der Zitate, der Zitate pro Jahr und des Anteils von Frauen als Erst- und Seniorautorin, wurden in Form von Häufigkeiten, Mittelwert, Median und Interquartilsbereich analysiert. Die Korrelation zwischen dem Impact-Faktor der Zeitschrift und dem Anteil von Frauen in Zeitschriften mit hohem Impact-Faktor wurde ermittelt. Ein p-Wert von <0,05 wurde als signifikant angesehen.

**Ergebnisse:**

Die meisten der am häufigsten zitierten Artikel wurden im Zeitraum 2001–2010 veröffentlicht (n=47). Die 3 am häufigsten zitierten Artikel stammten aus den USA. Von den 100 Artikeln waren Frauen in 46% der Artikel Erstautorin und in 28% der Artikel Letztautorin, was ein erhebliches Geschlechtergefälle widerspiegelt. Der Anteil von Frauen als Hauptautorin stieg jedoch allmählich von 25% (n=10/30) auf 52% (n=24/47) im Zeitraum 2001–2010 und auf 92% (n=12/13) im Zeitraum 2011–2020. Da die USA die höchste Anzahl an eingeschlossenen Artikeln (n=48) aufwiesen, waren Frauen in 56% (n=27) der Artikel Hauptautorin. Der geringste Frauenanteil wurde in Österreich, Dänemark, Japan, den Niederlanden, Neuseeland, Nigeria, Portugal und UK beobachtet.

**Schlussfolgerung:**

Frauen sind in der veröffentlichten Literatur über die Untersuchung von lebensmittelbedingten Krankheitsausbrüchen unterrepräsentiert. Obwohl der Anteil von Frauen als Hauptautorin in letzter Zeit zugenommen hat, gibt es immer noch Ungleichheiten auf der Ebene der leitenden Autoren und Autorinnen, die eine Förderung von Frauen in der akademischen Wissenschaft erfordern.

## Introduction

The representation of women in the medical field has gradually increased over the past few decades [1]. However, there is still a wide gender gap concerning the inclusion of women in research [2]. The resultant under-representation of women as authors in medical literature adversely affects women’s professional success [3]. It is therefore crucial to analyze the published literature from a gender perspective. Bibliometric analysis best serves this purpose.

Bibliometric analysis is a method used to evaluate the characteristics, frequency, and pattern of citations in the available literature. This is an important yet easy-to-use technique. By analyzing the characteristics and trends of publications, bibliometric analysis helps to focus on neglected areas and draw comprehensive conclusions based on the existing evidence. 

Food-borne diseases pose a global public-health challenge. The scale of food-borne illnesses is so high, that one in every 10 individuals world-wide becomes ill due to consumption of contaminated food [4]. The published literature on food-borne pathogen outbreaks has not yet been analyzed from the gender perspective. This study aims to provide gender distribution in authorship in the 100 most-cited articles on food-borne disease outbreaks.

## Methods

The Scopus database was used to conduct this bibliometric analysis. Scopus has proven to be a wider database than PubMed and Web of Science when it comes to scientific literature [5], [6]. Two reviewers were selected to search the database during February 2023. The first list comprised original articles published between 1990 and 2020. The original articles were separated from the review articles using Scopus filters. We included all the articles on food-borne disease outbreaks involving field investigation and for which complete author information was available, including their names, gender, and country of origin. The review articles, guidelines, and those articles for which citation information was not available were excluded from the analysis.

The selected articles were related to food-borne disease outbreaks. The appropriateness and relevance were assessed by thoroughly examining the abstracts. In certain cases where abstracts were unavailable, we used other sources to obtain abstracts and determine their suitability based on our inclusion criteria. The “cited by” filter in Scopus was used to arrange the articles in order of citations. We finally compiled the list of the 100 most-cited original articles. Only those articles were included to which both the reviewers agreed.

The analysis of the final list of articles was conducted using Statistical Package for Social Sciences (SPSS) version 26 and Microsoft Excel version 2016. The citation data including total citations, citations per year, and representation of women as first and senior authors was analyzed in terms of frequencies, mean, median, and interquartile range. Microsoft Excel was used to visualize data through appropriate graphs and tables. The authors’ information was extracted to determine the nationality and gender of the first and senior authors. We used SPSS to determine the correlation between journal impact factor and the representation of women in high-impact–factor journals in the 100 most-cited articles. A p-value of <0.05 was considered significant. 

## Results

Table 1 (Tab. 1) shows the list of top-cited articles in descending order. The top 3 most-cited articles – namely, “An Outbreak of Diarrhea and Hemolytic Uremic Syndrome From Escherichia coli O157:H7 in Fresh-Pressed Apple Cider”, “An Outbreak of toxic encephalopathy caused by eating mussels contaminated with domoic acid”, and “A large community outbreak of salmonellosis caused by intentional contamination of restaurant salad bars” – were all from the USA. 

### Distribution of articles over time

**Figure 1:**
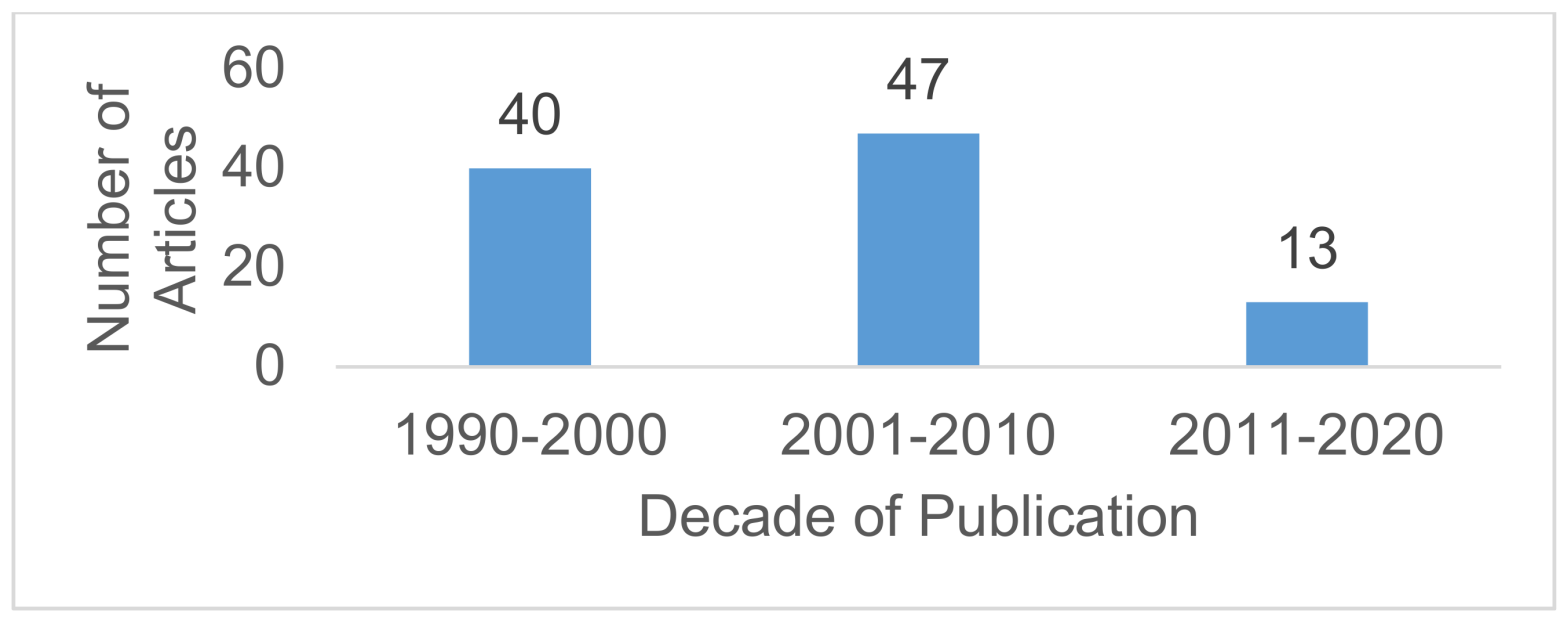
Distribution of published articles over the three decades

Figure 1 (Fig. 1) shows the distribution of published articles over the three decades from 1990 to 2020. The articles were grouped according to their year of publication into three groups: 1990–2000, 2001–2010, and 2011–2020. Most of the top-cited articles were published from 2001 to 2010 (n=47).

### Country-wise distribution of the most-cited articles

**Figure 2:**
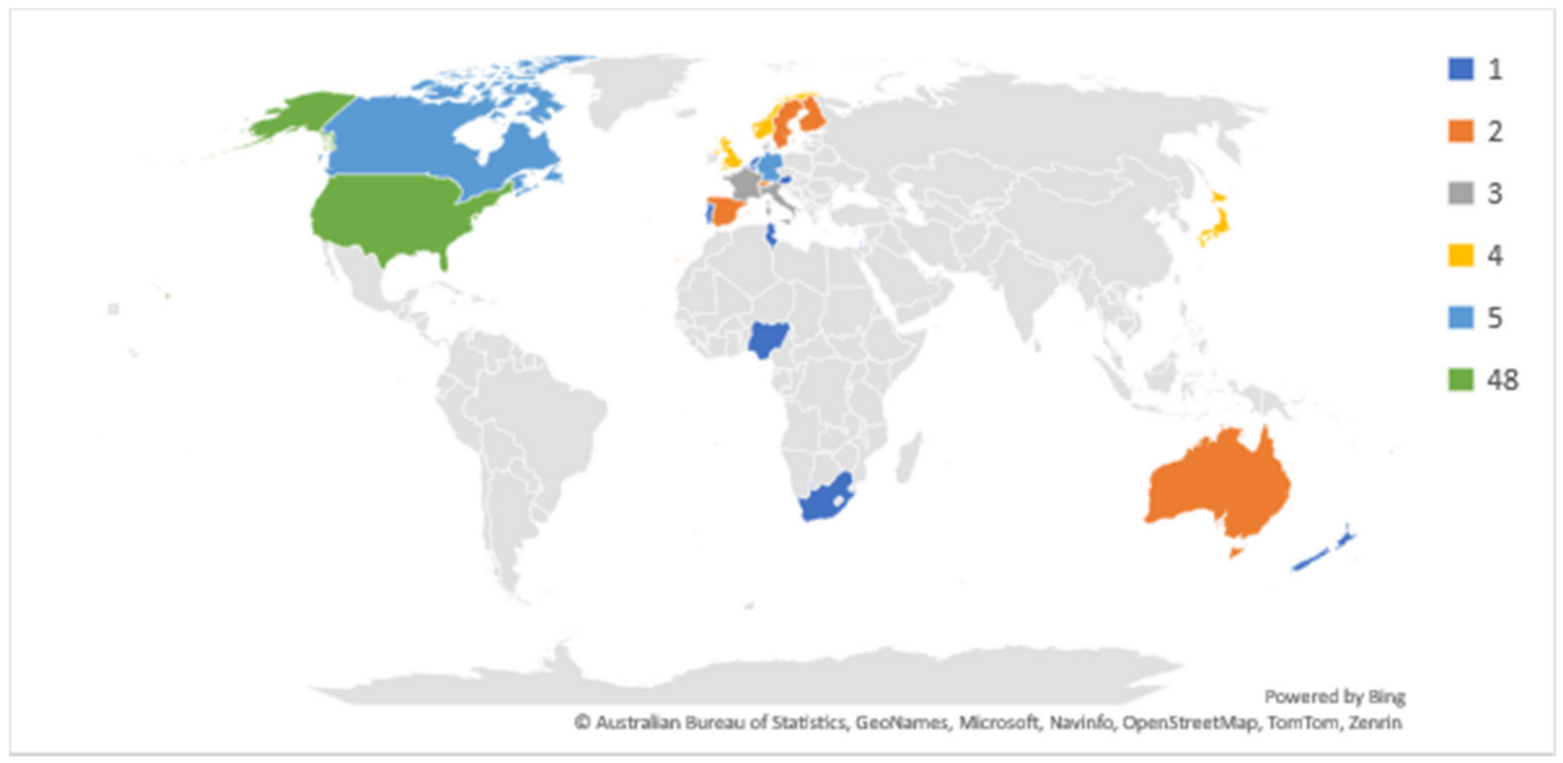
Country-wise distribution of the most cited articles

Figure 2 (Fig. 2) shows the country-wise distribution of the most cited articles. Most of the articles were from USA (n=48) followed by the UK and Canada (n=5). 

### Gender distribution in authorship

Of the total 100 articles, women were first and last authors in 46% and 28% of the articles, respectively. In contrast, 54% and 71% of the first and last authors, respectively, were male. This reflects significant gender disparity, as women were less represented in most of the most-cited articles. However, we noted a temporal trend towards improvement in the representation of female first and last authors over the years. The proportion of females as principal investigators has gradually increased from 25% (n=10/30) to 52% (n=24/47) during 2001–2010 and to 92% (n=12/13) during 2011–2020.

### Country-wise representation of women as principal author

**Figure 3:**
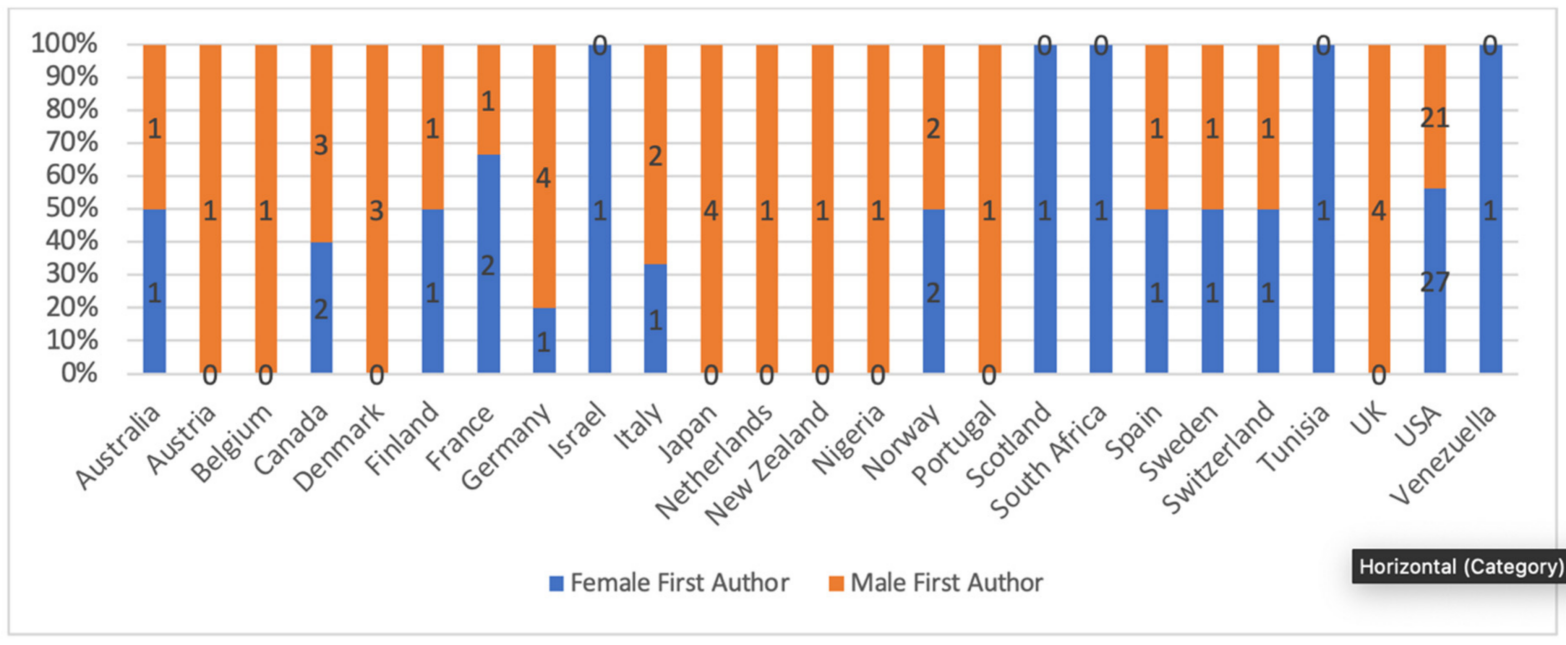
Country-wise representation of women as principal authors

Figure 3 (Fig. 3) shows a country-wise representation of women as principal authors. Israel, Scotland, South Africa, Tunisia, and Venezuela had the highest representation of women (100%) as principal authors. However, there was only one article from all these countries in the 100 top-cited articles. The USA had the highest number of included articles (n=48), and women were principal authors in 56% (n=27) of the articles. The lowest representation of women was observed in Austria, Denmark, Japan, Netherlands, New Zealand, Nigeria, Portugal, and UK where women authored none of the included articles as a principal investigator.

## Discussion

We conducted the bibliometric analysis to evaluate authorship trends by gender for the most-cited articles on food-borne disease outbreaks over the last three decades. We showed that, despite a temporal trend towards improved female representation in the specialty of food-borne disease-outbreak investigations, female authors continue to be a minority among top-performing articles published on this subject over the years. This data corroborates previously conducted studies which demonstrated the under-representation of female authors in various medical specialties ranging from pathology to epidemiology to psychiatry to critical care to surgery. 

According to UNESCO Institute for Statistics (UIS) data from 2016, less than 30% of STEM (science, technology, engineering and mathematics) researchers are female [7]. According to a study from the USA, female authors accounted for only 32.2% in the pathology clinical practice guidelines [8]. In 2018, the representation of female first authors in the field of emergency medicine was only 30% [9]. From 1987 to 2017, women orthopedic surgeons made up only 1.7% of senior writers and authors of orthopedic papers [10]. The same trend was apparent in the cardiology [11] and neurosurgery [12] literature, where female first authors made up only 20.8% and 10.5% of the total, respectively. 

This gender disparity in the research output on food-borne disease outbreaks can be attributed to several factors. Firstly, females face implicit bias and social barriers during recruitment into a laboratory for graduate research, acceptance for postdoctoral positions, recruitment to fill tenure-track faculty positions, and evaluation for promotion in rank [13]. For instance, there are only 38% female pathologists and 37% female public health and preventive specialists in the US [14]. Although these constraints have been gradually lifting, the consequences are still evident in the under-representation of women in senior medical positions and as authors. Secondly, there are further gender differences in grant funding. Even when women’s research is funded, their performance and research accomplishments as principal investigators are likely to be judged harsher than those of their male counterparts [15]. 

Thirdly, according to some studies, there may be a bias in the publication process that favors work by male authors. This can result in women’s research being published less frequently or in journals with lower impact factors, reducing their visibility and career advancement [16]. Such bias could undermine women’s greater research productivity by preventing publication of their submitted papers. Moreover, women may have difficulties in developing collaborative networks because of implicit biases or exclusion from informal networks that frequently lead to more publication opportunities. Finally, having effective mentors and role models can have a significant impact on an individual’s career path. Female students and early-career professionals may lack the necessary assistance and direction in the medical field, where women are under-represented in leadership roles [13].

Our analysis shows that there is a trend toward increasing involvement of female authors in the field of outbreak investigation, i.e., a 67% increase in 2020 from 1990, which seems promising. Nonetheless, despite significant progress, a gender disparity persists, notably among senior faculty and leadership, as there was no increase in the number of female senior authors from 1990. This raises a concern that even in fields where women’s representation is increasing at lower ranks [17], they do not have equal representation at higher levels. Gendered divisions of labor within academia may be to blame for this imbalance. This disparity shows that the problem will not be solved just by more generations of women entering the academic pipeline, but that women’s advancement in academic science must be fostered. 

### Limitations

There are some limitations to the current analysis. First, the gender of the authors was identified by inspecting first names and conducting internet searches, and hence there may have been some inaccuracy in gender assignment. Second, because this analysis included only the top 100 hundred referenced publications, the results may not truly represent the total food-borne disease-outbreak literature. Third, our data solely show the representation of female writers in published journal articles, without looking into whether there is any gender prejudice in the acceptance or rejection of female first- or second-authored manuscripts.

## Notes

### Competing interests

The authors declare that they have no competing interests.

### Author’s ORCID

- Mohsan Ali : 0000-0002-5697-0458

## Figures and Tables

**Table 1:**
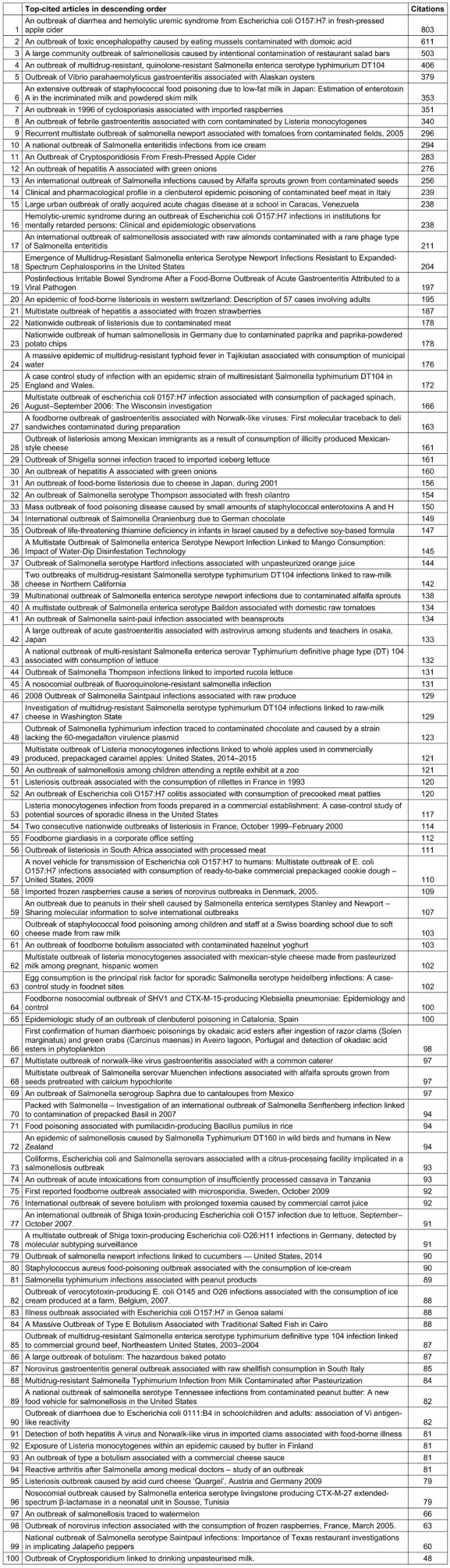
Top-cited articles in descending order

## References

[1] Kang SK, Kaplan S (2019). Working toward gender diversity and inclusion in medicine: myths and solutions. Lancet.

[2] Silver JK, Ghalib R, Poorman JA, Al-Assi D, Parangi S, Bhargava H, Shillcutt SK (2019). Analysis of Gender Equity in Leadership of Physician-Focused Medical Specialty Societies, 2008-2017. JAMA Intern Med.

[3] Beasley BW, Simon SD, Wright SM (2006). A time to be promoted. The Prospective Study of Promotion in Academia (Prospective Study of Promotion in Academia). J Gen Intern Med.

[4] World Health Organization (WHO) (2022). Food safety.

[5] AlRyalat SAS, Malkawi LW, Momani SM (2019). Comparing Bibliometric Analysis Using PubMed, Scopus, and Web of Science Databases. J Vis Exp.

[6] Pranckutė R (2021). Web of Science (WoS) and Scopus: The titans of bibliographic information in today’s academic world. Publicat.

[7] UNESCO Institute of Statistics (2020). Women in science.

[8] Martin AA, Walker SC, Wheeler AP, Jacobs JW, Booth GS, Silver JK (2023). Representation of Authors by Gender, Race, and Ethnicity in Pathology Clinical Practice Guidelines. Arch Pathol Lab Med.

[9] Webb J, Cambron J, Xu KT, Simmons M, Richman P (2021). First and last authorship by gender in emergency medicine publications- a comparison of 2008 vs. 2018. Am J Emerg Med.

[10] Brown MA, Erdman MK, Munger AM, Miller AN (2020). Despite Growing Number of Women Surgeons, Authorship Gender Disparity in Orthopaedic Literature Persists Over 30 Years. Clin Orthop Relat Res.

[11] Asghar M, Usman MS, Aibani R, Ansari HT, Siddiqi TJ, Fatima K, Khan MS, Figueredo VM (2018). Sex Differences in Authorship of Academic Cardiology Literature Over the Last 2 Decades. J Am Coll Cardiol.

[12] Aslan A, Kuzucu P, Karaaslan B, Börcek AÖ (2020). Women in Neurosurgery: Gender Differences in Authorship in High-Impact Neurosurgery Journals through the Last Two Decades. World Neurosurg.

[13] National Academies of Sciences, Engineering, and Medicine, Policy and Global Affairs, Committee on Women in Science, Engineering, and Medicine, Committee on Increasing the Number of Women in Science, Technology, Engineering, Mathematics, and Medicine (STEMM), Helman A, Bear A, Colwell R, (2020). Factors that Drive the Underrepresentation of Women in Scientific, Engineering, and Medical Disciplines.

[14] Statista (2023). Share of female physicians in select specialties in the U.S. as of 2022 (n.d.).

[15] Witteman HO, Hendricks M, Straus S, Tannenbaum C (2019). Are gender gaps due to evaluations of the applicant or the science? A natural experiment at a national funding agency. Lancet.

[16] Filardo G, da Graca B, Sass DM, Pollock BD, Smith EB, Martinez MA (2016). Trends and comparison of female first authorship in high impact medical journals: observational study (1994-2014). BMJ.

[17] Carr PL, Gunn C, Raj A, Kaplan S, Freund KM (2017). Recruitment, Promotion, and Retention of Women in Academic Medicine: How Institutions Are Addressing Gender Disparities. Womens Health Issues.

